# Foreign Body in the Nasal Cavity: A Case Report

**DOI:** 10.7759/cureus.50373

**Published:** 2023-12-12

**Authors:** Mohammed Asiri, Mohammed S Al-khulban, Ghalib Al-Sayed

**Affiliations:** 1 Otorhinolaryngology-Head and Neck Surgery, Aseer Central Hospital, Abha, SAU; 2 Otolaryngology-Head and Neck Surgery, Armed Forces Hospital Southern Region, Khamis Mushait, SAU

**Keywords:** case report, impacted foreign body, foreign body insertion, pediatric patient, nasal foreign body

## Abstract

We present a case of a pediatric patient who presented to the emergency room with acute nasal discharge, foul smell, and nasal pain. The patient's mother witnessed her inserting a foreign body into the nasal passage. After thorough examination and diagnostic imaging, a metallic necklace bead was identified as the foreign body lodged in the nasal cavity. The patient was promptly prepared for emergency operating room intervention. The metallic foreign body was successfully extracted without complications using endoscopic equipment and careful manipulation. The patient recovered well, was admitted for one day for observation and supportive care, and was discharged home in excellent condition. Follow-up in the clinic revealed a satisfied patient with no complaints, no septal perforation, and a patent airway.

## Introduction

Foreign body insertion into the nasal cavity is a common pediatric emergency, often necessitating prompt intervention to prevent complications [1,2]. It is more prevalent among toddlers due to their behavior, curiosity, and exploration of their surroundings [2]. Detecting these foreign bodies can be a complex task due to the presence of non-specific symptoms, the absence of a clear patient history, or a combination of these factors [2,3]. In some cases, foreign bodies may be aspirated from the nasal cavity, leading to aspiration symptoms [4,5]. Utilizing imaging techniques is pivotal in the identification of aspirated foreign bodies in pediatric patients and can be instrumental in directing the clinical treatment of these individuals [2]. Previous studies found that foreign body aspiration in children exhibited an uneven distribution, with the highest occurrence noted among children residing in rural areas (70%). The age group most significantly affected was 1-3 years, encompassing 79% of all cases, and the primary symptoms that led to urgent hospital admission were coughing (33%) and dyspnea (22%) [5].

Prompt recognition and intervention, along with a tailored approach to each case, are key to ensuring a positive outcome for the child. Therefore, here, we present a case of a pediatric patient who presented to the emergency room with acute symptoms resulting from a metallic foreign body lodged in the nasal cavity. We describe the diagnostic process, successful extraction, and post-operative management, which could guide healthcare professionals in their efforts to address this prevalent issue and improve pediatric care.

## Case presentation

A five-year-old female child with no significant medical history presented to the emergency room with a one-day history of nasal discharge, foul smell, and pain in the left nostril. Her mother witnessed her inserting a foreign body into the nose. On examination, the patient appeared anxious and in mild distress. No foreign body was visible within the nasal cavity, but adenoid hypertrophy grade 3 was noted using a fiberoptic endoscope. Additionally, bilateral inferior turbinate hypertrophy was observed, and there was watery mucous discharge from the left nostril.

**Figure 1:**
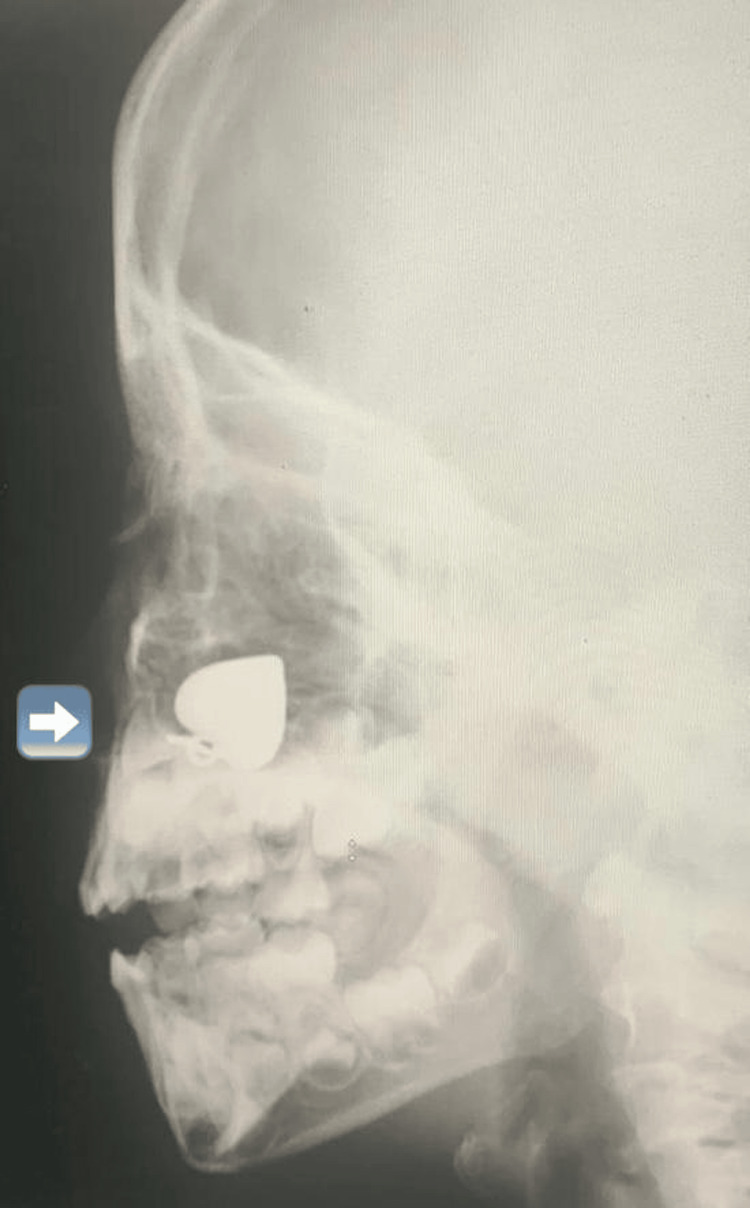
X-ray showing a metallic spade-shaped foreign body in the nasal cavity

An X-ray was obtained, which revealed the presence of a spade-shaped metallic foreign body in the nasal cavity (Figure 1), confirming the mother's observation. Given the urgency of the situation, the patient was promptly prepared for the operating room (OR).

**Figure 2:**
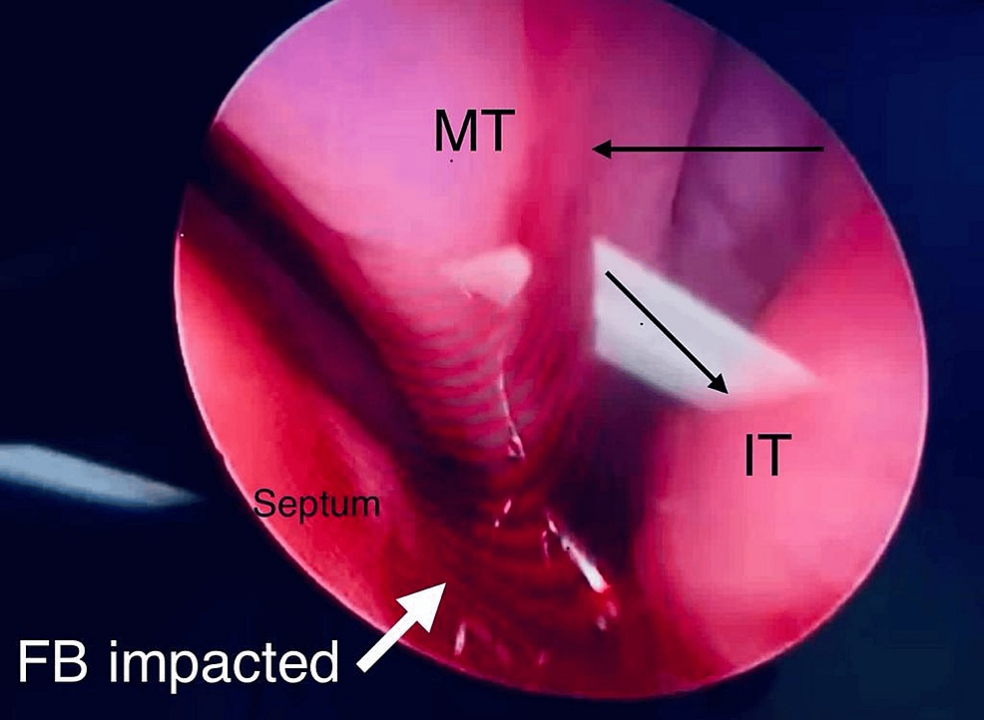
Endoscopic view of the metallic foreign body impacted posteriorly in the inferior nasal passage FB: Foreign body; MT: Middle turbinate; IT: Inferior turbinate

**Figure 3:**
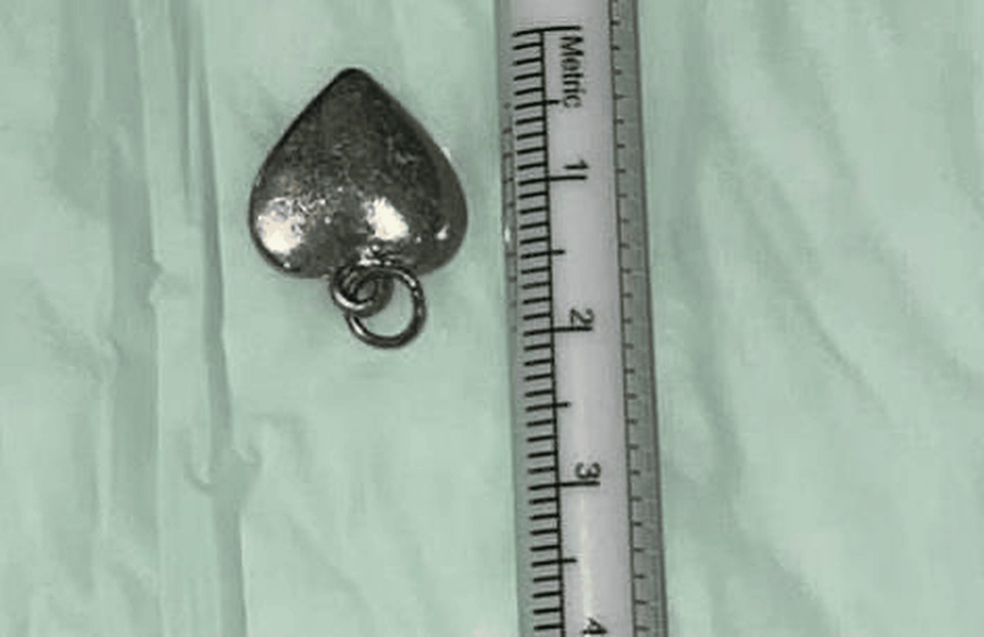
A spade-shaped metallic necklace bead extracted

In the OR, the patient received nasal packing with xylometazoline for five minutes to minimize bleeding and cause vasoconstriction to facilitate manipulation. A 0-degree endoscopic scope was utilized for visualization. Artery forceps were used to grasp the metallic foreign body, which was found to be impacted posteriorly in the inferior nasal passage (Figure 2). Careful manipulation allowed for the successful extraction of the spade-shaped metallic necklace bead (Figure 3), and the patient was smoothly extubated and transferred to the recovery room in good condition.

The patient was admitted for observation for one day post-operatively and received antibiotics, analgesics, and xylometazoline to prevent infection and manage any residual discomfort. During the hospital stay, the patient remained afebrile, and there was no evidence of bleeding.

The patient was discharged home in excellent condition and returned for a follow-up clinic visit. At the follow-up appointment, the patient and her parents expressed satisfaction. There were no complaints, and a thorough examination revealed no septal perforation or synechiae. The patient had a patent airway, and the outcome was deemed successful.

## Discussion

Pediatric nasal foreign body insertions are common among toddlers and young children and can present with a variety of symptoms [1,6]. In our case, the pediatric patient successfully underwent the removal of a metallic foreign body from the nasal cavity with endoscopic equipment in the operating room. A previous study on 87 children by Aslan et al. found that 42.5% were of the female gender [7]. Though in the majority of cases (62.1-71%), the foreign body was found to be organic in nature [7,8], the round-shaped foreign bodies, whether organic or inorganic, are the most commonly found inserted in the nasal cavities by children [9]. Our patient inserted a spade-shaped foreign body, which has round borders, making it easier to insert.

Age is a significant risk factor, with the highest incidence occurring in toddlers and young children. This can be attributed to their developmental stage, curiosity, and limited understanding of the consequences of their actions. Hira et al. reported that the mean age of children with nasal foreign bodies was 3.3 years, with a peak incidence in the 2 to 3-year age group [6]. Additionally, children with certain behavioral or psychiatric disorders may be at an increased risk. Conditions such as attention deficit hyperactivity disorder (ADHD) or developmental delays can result in impulsive behaviors, making these children more prone to inserting foreign bodies into their nasal and aural cavities [10,11]. It was found that children with ADHD are three times at higher risk of nasal foreign body self-insertion [11]. Therefore, understanding these risk factors can help healthcare professionals and caregivers be more vigilant in high-risk situations.

The causes of nasal foreign body insertions in children are varied and can be divided into three categories: accidental, exploratory, and behavioral. Accidental insertions can happen as a result of a fall, an accident, or while playing with things. Unaware of the dangers, children may insert small objects into their noses [1]. Exploratory insertions are frequently the consequence of children's natural curiosity. They may insert objects into their nostrils as a form of entertainment, experimentation, or imitation. Little objects, such as beads, food, or little toys, are popular alternatives for children to investigate [6,9,12]. This is similar to our patient who inserted a necklace bead, highlighting the need for extra vigilance for her parent, who reported seeing her inserting something. Parents and carers should be on the lookout for indications and symptoms of nasal foreign body insertions, such as unilateral nasal discharge, foul smell, pain, or bleeding [1,13], most of which were observed in our case.

The treatment of nasal foreign bodies in children depends on several factors, including the nature and location of the foreign body, the child's cooperation, and the experience of the healthcare provider. Conservative measures, such as asking the child to blow their nose gently or using techniques like the parent's “kiss" to remove the object, may be attempted in cooperative children [14,15]. Though the parent’s kiss technique is successful in 64.5% of cases, it may inadvertently push the foreign body deeper into the nasal passage [15,16], requiring surgical intervention. For more complex cases, medical professionals often employ specialized instruments or endoscopic equipment to visualize and extract the foreign body. In uncooperative or fearful children, sedation or general anesthesia may be necessary to ensure a safe and efficient procedure. Studies have shown that endoscopic removal is a safe and effective method associated with high success rates and minimal complications [12,17], which is the method used in our case, and it was successful without any complications. The choice of intervention may also depend on the type of foreign body. Sharp or potentially hazardous objects may necessitate prompt and more invasive treatment to prevent injury [18]. 

When managed appropriately, the majority of cases result in complete recovery, with children returning to their normal activities without lasting complications. However, the prognosis can be influenced by several factors, including the promptness of intervention, the type and location of the foreign body, and any associated trauma or injury. Early removal is associated with improved outcomes and reduced risks of complications [1,11]. Delays in diagnosis and treatment can result in complications such as local infections, nasal septal perforation, chronic nasal obstruction, epistaxis, rhinosinusitis, rhinolith, aspiration, and severe infection [8,13]. The long-term complication is synechiae formation [11,13].

Chronic nasal obstruction can result from septal deviation, synechiae, or granulation tissue formation, which may require further intervention [19,20]. The absence of these complications in our case indicates that early and effective management is crucial in preventing these complications and ensuring a favorable prognosis.

## Conclusions

In this case report, we presented a successfully managed child with nasal foreign body insertion without any complications. This underscores the need for a thorough understanding of the case, including the prevalence, risk factors, causes, treatment modalities, and prognosis for appropriate management. It also shows that prompt recognition and intervention are essential in ensuring a positive outcome for the child.
